# Blunted Expansion of Regulatory T Lymphocytes Is Associated With Increased Bacterial Translocation in Patients With Major Depressive Disorder

**DOI:** 10.3389/fpsyt.2020.591962

**Published:** 2021-01-08

**Authors:** Miguel Angel Alvarez-Mon, Ana Maria Gomez-Lahoz, Arantxa Orozco, Guillermo Lahera, M. Dolores Sosa-Reina, David Diaz, Agustin Albillos, Javier Quintero, Patricio Molero, Jorge Monserrat, Melchor Alvarez-Mon

**Affiliations:** ^1^Department of Psychiatry and Medical Psychology, University Clinic of Navarra, Pamplona, Spain; ^2^Department of Medicine and Medical Specialities, University of Alcala, Alcala de Henares, Spain; ^3^Department of Psychiatry and Mental Health, Hospital Universitario Infanta Leonor, Madrid, Spain; ^4^Department of Psychiatry, University Hospital “Principe de Asturias”, Alcalá de Henares, Spain; ^5^CIBERSAM (Biomedical Research Networking Centre in Mental Health), Madrid, Spain; ^6^Department of Gastroenterology, University Hospital Ramon y Cajal, Madrid, Spain; ^7^Institute Ramon y Cajal for Health Research (IRYCIS), Madrid, Spain; ^8^Biomedical Institute for Liver and Gut Diseases (CIBEREHD), Madrid, Spain; ^9^Department of Legal and Psychiatry, Complutense University, Madrid, Spain; ^10^Service of Internal Medicine and Rheumatology, Autoimmune Diseases University Hospital “Principe de Asturias”, Madrid, Spain

**Keywords:** major depressive disorder, regulatory T lymphocytes (Treg), chemorreceptors, LPS-binding protein, gut barrier, CD4+ lymphocytes, interleukin 10 (IL10)

## Abstract

**Background:** Major Depressive Disorder (MDD) is associated with both proinflammatory and adaptive immune response abnormalities. Regulatory T lymphocytes (Tregs), a subtype of CD4+ T cells, are relevant for maintaining immune-inflammatory system homeostasis and control of inflammation such as the kind potentially induced by the interactions between the intestinal microbiome and gut mucosa. We investigated the Treg population and its distribution along their stages of differentiation/activation, as well as its function in MDD patients without concomitant diseases. We also studied the potential association between Treg alterations, intestinal barrier damage, and bacterial translocation.

**Methods:** 30 MDD patients and 20 healthy controls were studied. The levels of circulating CD25FoxP3^+^ Tregs and their distribution on the naïve (T_N_), effector (T_E_), central (T_CM_), and effector memory(T_EM_) differentiation/activation stages were analyzed using polychromatic flow cytometry. Chemokine receptors (CCR) 2, 5, and 6, and the intracytoplasmic IL-10 expression by the Tregs were also analyzed. The serum IL-10 was measured using Luminex. The serum levels of zonulin and the intestinal fatty acid-binding protein (I-FABP), both markers of gut barrier function, and the LPS-binding protein (LBP), a marker of bacterial translocation, were measured using an enzyme-linked immunosorbent assay.

**Results:** MDD patients had increased number of circulating Tregs cells with enhanced number of Tregs at the T_N_, T_E_, T_CM_, and T_EM_ stages. The percentage of Tregs cells at T_N_ stage was significantly higher in MDD patients. The percentage of Tregs that expressed CCR2 and CCR6 was increased as well as those expressing IL-10. MDD patients had significantly increased levels of circulating I-FABP and LBP. MDD patients with high LBP levels had a significant reduction in the number of circulating Tregs compared to normal-LBP MDD patients.

**Conclusions:** MDD patients showed an expansion of circulating Tregs and their CD25^high^FoxP3^+^ and CD25^low^FoxP3^+^ subsets throughout the different stages of CD4+ T lymphocyte differentiation/activation. Tregs also showed an increased frequency of cells expressing CCR6 and CCR2. IL-10 Treg production was also enhanced in MDD patients that concurrently had increased serum IL-10 levels. However, this Treg expansion was blunted in MDD patients with gut barrier damage and increased bacterial translocation.

## Introduction

Major depressive disorder (MDD) is a disease with high prevalence. The lifetime risk of depression is 15–18%, meaning that almost one in five people will experience at least one episode at some point in their lifetime (1). It is considered to be the third leading cause of disability worldwide (2). There is increasing interest in developing a more effective MDD treatment in order to improve the poor response experienced in approximately a third of the patients (3). Many neurobiological systems have been implicated in the etiopathogenesis and pathophysiology of MDD (1). New therapeutic approaches that target MDD pathogenic mechanisms beyond monoamine modulation are needed, in part, because of the shortcomings of selective serotonin reuptake inhibitors, serotonin-norepinephrine reuptake inhibitor, and tricyclic antidepressant treatments.

Experimental and human data have highlighted the importance of aberrant immune-inflammatory response in the development of depression (4). The main finding supporting the abnormal function of the immune system in MDD patients is the consistency among articles reporting increased circulating levels of proinflammatory cytokines (5). The risk of depression is increased in many disorders with an inflammatory component (6–9). Much of the research has focused on the activation of the innate immune system (10). However, intriguing new evidence suggests that the disease profile of MDD patients includes an immunosuppressive component, especially when involving the adaptive immune system (11). This pathogenic perspective is supported by epidemiological data of greater susceptibility to viral infections, reduced immune responses to vaccines and the slowed wound healing in MDD patients (12–14). In addition, depressed individuals with infectious diseases and tumors show a worse prognosis (15, 16). The understanding of the cellular mechanisms involved in the pathogenesis of the MDD immune dysfunction remains partially understood.

Regulatory T lymphocytes (Tregs) are a subtype of CD4+ T cells known to be relevant for maintaining immune system homeostasis and self-tolerance (17). The Forkhead box P3 (FoxP3) transcription factor and the alpha chain of the IL-2 receptor (CD25) are the main markers of Tregs (18). Treg cells are a heterogeneous population based on their differentiation, phenotype, functional activity, and activation status (19). Depending on the intensity of CD25 expression two Tregs subsets can be identified, those with high or low expression (CD25^hi^FoxP3^+^ and CD25^low^FoxP3^+^ Tregs, respectively) with different commitment to Treg cell fate (20, 21). Two CD25^hi^FoxP3^+^ and CD25^low^FoxP3^+^ Tregs subsets may be identified by the intensity of CD25 expression known as “resting” and effector Treg cells.

Tregs are highly heterogeneous (22). One subdivision is based on the history of antigen activation that distinguishes naïve (T_N_) Tregs from effector (T_E_), central (TCM), and effector memory (TEM) Tregs that can be identified by the expression of the RO and RA isoforms of the CD45 common leukocyte antigen family and the CCR7 antigen (23). These Treg subsets have different distinctive patterns of activation and effector functions (22). In summary, the T_N_ Treg subset exhibits a non-effector function while T_CM_ Tregs can rapidly proliferate and express effector molecules after being stimulated by an antigen and diminished activation requirements. T_EM_ Tregs produce effector cytokines but have limited proliferative capacity while T_E_ Tregs are at a final differentiation stage and possess high levels of cytokine production. Tregs can exert their immune suppression capacity by utilizing different mechanisms. The production of interleukin (IL) 10 by Tregs is a pivotal mechanism for inhibition of cells of the innate and adaptive immune responses (24). Tregs display different homing properties, and their appropriate compartmentalization is crucial for their *in vivo* activity. Several chemokine receptors (CCR) regulate Tregs tissue traffic including the migration to inflamed tissues (25). For instance, loss of CCR6 prevents directing Treg migration to inflamed tissues whereas CCR5 expression helps in directing Treg migration to inflamed tissues. Abnormalities in CCR 2, 5, and 6 expression by Tregs have shown to have pathogenic relevance in different inflammatory disorders (26).

Quantitative and functional alterations of Tregs have been implicated in the development of several common autoimmune and inflammatory diseases such as systemic lupus erithematosus and rheumatoid arthritis (27). The pathogenic relevance of Tregs has also been demonstrated in patients with cancer or infectious diseases. The potential alteration of Tregs in MDD patients has been investigated but contradictory numbers (increased, normal, and decreased) of Tregs have been reported (28–32).

Tregs play a key role in the control of harmful inflammation such as the kind potentially induced by the interactions between the intestinal microbiome and gut mucosa (33). Damage of the intestinal barrier with increased gut permeability and bacterial translocation has been found in MDD patients (34). Furthermore, increased levels of the LPS-binding protein (LBP), a bacterial translocation biomarker, have been associated with a higher systemic proinflammatory stage and monocyte activation in MDD patients (35).

Along these lines, we hypothesized that a heterogenous Treg alteration in MDD patients could explain the contradictory results previously described. We also investigated a potential association of gut barrier damage and bacterial translocation with Treg alterations in MDD patients. We considered it critical that the phenotypical and functional study of Tregs and gut barrier permeability had to be performed in MDD patients without concomitant diseases that could otherwise have potential interactions with the immune system or with the gut barrier.

Our work focused on the study of a homogeneous population of 30 patients with refractory MDD and no concurrent diseases that could be associated with immune system abnormalities. The patients were not homogenous in terms of pharmacological treatment. In parallel, we analyzed 20 age- and sex-matched healthy controls (HCs). We studied the numbers of T_N_, T_E_, T_CM_, and T_EM_ of Tregs along with their CD25^hi^FoxP3^+^ and CD25^low^FoxP3^+^ subsets. We also analyzed the pattern of CCR 2, 5, and 6 receptor expression and IL-10 production by activated Tregs, as well as the serum levels of IL-10. In addition, the serum levels of zonulin and the intestinal fatty acid-binding protein (I-FABP), both markers of gut barrier function, and the LPS-binding protein (LBP), a marker of bacterial translocation, were also measured.

## Materials and Methods

### Patients and Study Protocol

In this study, we included 30 patients with MDD from the Department of Psychiatry of the Clinica Universidad de Navarra and from the Hospital Universitario Príncipe de Asturias. The inclusion criteria included the following: (a) psychiatrist-confirmed diagnosis of MDD, single or recurrent, according to the Diagnostic and Statistical Manual of Mental Disorders criteria, Fifth Edition (DSM-V) (36); (b) a minimum score of 14 points on the 17-item Hamilton Rating Scale for Depression (HRSD); and (c) age, 18–65 years. Potential subjects were excluded for the following reasons: (1) acute infection in the last 3 months; (2) chronic bacterial or viral infection; (3) the use of steroids or immunomodulatory drugs in the last 3 months; (4) an autoimmune disease; (5) a cardiovascular disease, including hypertension and ischemic heart disease; (6) a hematopoietic, lung, hepatic, or renal disorder; (7) an endocrine or metabolic disease, including diabetes mellitus and hypercholesterolemia, or a body-mass index (BMI) higher than 30; (8) a history of malignancy; (9) immunodeficiency and malnutrition; (10) pregnancy or lactation; and (11) concomitant psychiatric illness, assessed with the MINI International Neuropsychiatric Interview (37). The patients with refractory MDD included in the suty were not homogenous in terms of psychotropic drugs on board. The patients were studied in parallel with 20 sex-, age-, and BMI-matched HCs from the same epidemiological area.

This study was approved by the ethics committee of the University of Navarra and the Hospital Universitario Príncipe de Asturias. After the study procedures had been fully explained, the subjects provided written informed consent before study enrollment.

Blood samples were drawn from all subjects via standard venipuncture using an established aseptic technique. Samples were obtained at the time of the evaluation. Serum samples were also included for analysis. After collection, the samples were centrifuged, and the serum was isolated, aliquoted, and stored at −80°C until analysis.

### Isolation of Peripheral Blood Mononuclear Cells

Blood was collected by antecubital puncture from patients and healthy controls. Peripheral blood mononuclear cells (PBMC) were obtained from heparinized venous blood by Ficoll-Hypaque (Lymphoprep™, Axis-Shield, Oslo, Norway) gradient centrifugation. They were then resuspended in RPMI 1640 (Biowhittaker Products, Verviers, Belgium) supplemented with 10% heat-inactivated fetal calf serum, 25 mM Hepes (Biowhittaker Products) and 1% penicillin-streptomycin (Biowhittaker Products). Cell enumeration was performed by conventional light microscopy using a Neubauer chamber following trypan blue dead cell exclusion criteria The viability of fresh PBMC was checked by both trypan blue (light microscopy) and flow cytometry exclusion.

### *In vitro* Culture

Stimulated Tregs expression of IL-10 was assessed *in vitro* intro intracytoplasmic staining in the presence of brefeldin (38). T cells were stimulated with 50 ng/ml phorbol-12-myristate-13-acetate (PMA, Sigma Aldrich Quimica, Spain) plus 1 μg/ml ionomycin (Calbiochem-Novabiochem, La Jolla, CA) for 6 h. The study of regulatory T lymphocytes was determined in parallel at 4°C in the absence of exogenous stimuli.

### Surface and Intracellular Lymphocyte Staining

T cells were phenotypically analyzed in PBMC by nine-colors polychromatic in flow cytometry in a FACSAria cytometer using FACSDiva software (Becton-Dickinson). For surface staining, 1 million cells PBMC were incubated in four FACS tubes with monoclonal antibodies combinations of fluorescein IsoTioCyanate (FITC)-anti-CCR2/CCR5/CCR6 (Biolegend), peridinin chlorophyll protein (PercP)-anti-CD3 (Biolegend), phycoerythrin-cyanine seven (PE-Cy7)-anti-CD25 (BD), allophycocyanin-alexa-780 (APC-Alexa780)-anti-CCR7 (eBioscience), brilliant violet-405-anti-CD4 (Biolegend), and brilliant violet-605-anti-CD45RA (Biolegend). The cells were fixed and permeabilized with Fix and Perm solution (Anti-Human Foxp3 Set, eBioscience) during 35 min, and then, cells were washed with phosfate saline buffer (PBS) plus FBS (fetal bobine serum), and incubated with Permeabilitation Buffer and Normal Rat Serum (eBioscience) during 15 min. Finally, the cells were incubated 30 min with intracellular monoclonal antibodies: phycoerythrin (PE)-anti-IL-10 (Becton-Dickinson) and allophycocyanin (APC)-anti-FoxP3 (eBioscience).

### Quantification of Serum Proteins

To study the concentrations of IL-10 in the serum, the Milliplex MAP Kit (MERCK, Darmstadt, Germany) was employed using the protocol recommended by MERCK. The plate was read in a Luminex MAGPIX with xPONENT software. The concentration of the cytokine was calculated from the standard curve using mean fluorescence intensity (MFI) analysis with Analyst software (MERCK).

To study intestinal barrier damage, the concentration of I-FABP and zonulin in the serum were measured by enzyme-linked immunosorbent assay (ELISA). I-FABP was purchased from Hycult Biotech (Hycult Biotech, PA, USA), and zonulin was purchased from R&D Systems (R&D Systems, MN, USA). #e tests were carried out according to the protocols provided in the kits. #e plate was read in an iMark Microplate Reader at 450 nm with Microplate Manager So(ware (Termo Fisher Scientific, MA, USA).

To study bacterial translocation, the concentration of LBP in the serum was measured by ELISA (Abnova, Taipei, Taiwan). We performed 1:800 dilutions of the samples from the MDD patients and HCs. The test was carried out according to the protocol provided in the kit. The plate was read in an iMark Microplate Reader at 450 nm with Microplate Manager Software (Thermo Fisher Scientific).

### Statistical Analysis

Analyses were performed using SPSS-19 software (SPSS-IBM, Armonk, NY). Since most variables did not fulfill the normality hypothesis, the Mann Whitney *U*-test for non-parametric data was used to analyze differences between independent groups. Significance level was set at *p* < 0.05.

## Results

### Demographic Patient Characteristics

Table 1 shows the demographic data and clinical characteristics of the 30 MDD patients and 20 HCs included in the study. No significant differences were found between MDD patients and HCs with respect to the variables that were studied, except for employment status. The patient group included 19 females and 11 males, ranging from 27 to 53 years of age. The duration of their depressive episode before recruitment was 16.12 ± 2.85 weeks. Seventeen patients (56.7%) had suffered at least one previous MDD episode. At the time of the study, the mean value of the HRSD was 15.95 ± 1.25. Furthermore, 10% of the patients presented psychotic (delusional) symptoms during the current episode.

**Table 1:** Sample characteristics.

	**MDD**	**HC**	***p*-value**
**Socio-demographic**			
Age, mean (SD)	43.26 (13.14)	40.45 (12.46)	0.45
Sex (% female)	19 (63.3%)	12 (60%)	0.98
Currently employed and active *n* (%)	13 (59.1%)	18 (90%)	<0.01
College degree *n* (%)	16 (53.3%)	12 (60%)	0.77
**Past history**			
Family history of depression *n* (%)	17 (56.7%)	8 (40%)	0.38
Family history of other psychiatric disorder *n* (%)	22 (77.3%)	11 (55.5%)	0.22
**Health characteristics and somatic morbilities**			
BMI, mean (SD)	26.74 (5.41)	25.5 (5.36)	0.51
Smoking *n* (%)			0.13
Never	12 (40%)	6 (30%)	
Occasionally	8 (26.7%)	11 (55%)	
Everyday	10 (33.3%)	3 (15%)	
Drinking *n* (%)			0.43
Never	7 (23.3%)	3 (15%)	
Occasionally	20 (66.7%)	16 (80%)	
Everyday	3 (10%)	1 (5%)	

All patients received pharmacological treatment at their doctor's discretion: 30 (100%) received antidepressant medications, 28 (93.3%) received anxiolytics or hypnotics, 5 (16.7%) received mood stabilizers, and 10 (33.3%) received atypical antipsychotics. 28 (93.3%) received combination pharmacotherapy, consisting of at least 2 different types of medication in 19 patients (63.3%) and of at least 3 different types of medication in the other 9 patients (30%). None of the patients received electroconvulsive therapy (ECT).

### MDD Patients Show Increased Number of Circulating Tregs at the Different Stages of CD4^+^ Differentiation

**Figure 1:**
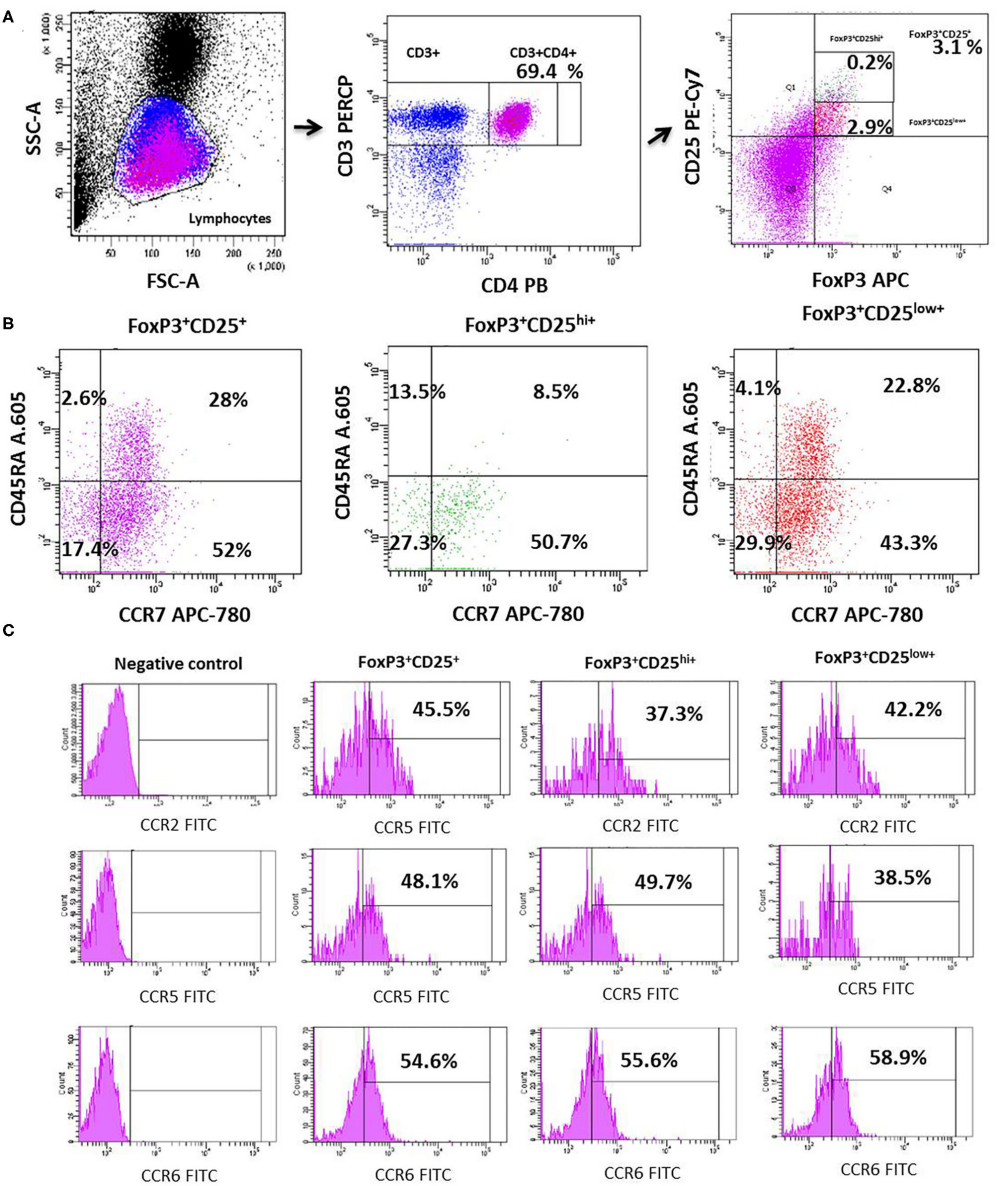
Dot plots represent the flow cytometry gating strategy and histograms of the circulating total Tregs and their CD25^high^FoxP3^+^ and CD25^low^FoxP3^+^ subsets in a representative case of MDD. The first row dot plots represent the selected gates and percentages to obtain the total Tregs **(A)**, the second those of the individualized total Tregs and their CD25^high^FoxP3^+^ and CD25^low^FoxP3^+^ subsets (**B**, respectively). The **(C)** shows dot plots of the selected gates and percentages of the CCR2 (superior line), CCR5 (medium line), and CCR6 (lower line) expression by total Tregs and their CD25^high^FoxP3^+^ and CD25^low^FoxP3^+^ subsets.

**Figure 2:**
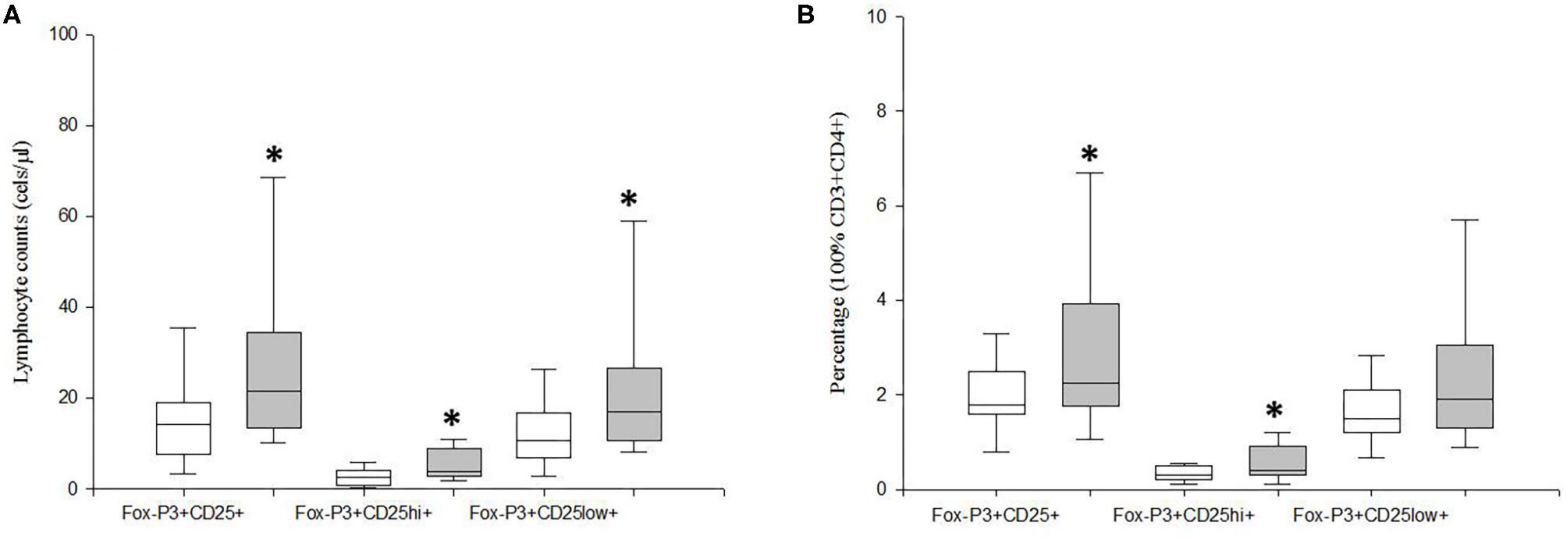
Absolute number and frequency of total circulating Tregs and their CD25^hi^FoxP3^+^ and CD25^low^FoxP3^+^ subsets in MDD patients and healthy controls. Absolute number (cells/μl) (y axis) of CD25^+^FoxP3^+^ lymphocytes and their CD25^hi^FoxP3^+^ and CD25^low^FoxP3^+^ subsets in MDD patients (gray box plots) and healthy controls (white box plots) are shown in panel **(A)**. Frequency of the total Tregs and their CD25^hi^FoxP3^+^ and CD25^low^FoxP3^+^ subsets in the circulating CD4+ T lymphocytes in MDD patients (gray box plots) and controls (white box plots) are shown in **(B)**. The top of the rectangle indicates the third quartile, the horizontal line near the middle of the rectangle indicates the median, and the bottom of the rectangle indicates the first quartile. A vertical line extends from the top of the rectangle to indicate the maximum value, and another vertical line extends from the bottom of the rectangle to indicate the minimum value. *Significant difference between MDD and healthy controls for the indicated variable.

We investigated the circulating counts of Tregs and their CD25^hi^FoxP3^+^ and CD25^low^FoxP3^+^ subsets defined by the level of CD25 expression in MDD patients and age-, sex-, and ethnically matched HCs. Figure 1 shows the flow cytometry gating strategy and histograms of Tregs in a representative case of MDD patient. We found a significant increase in the number of circulating Tregs cells in MDD patients with respect to HCs (*p* = 0.01) (Figure 2). This Tregs expansion is explained by a significant increase in the counts of both CD25^hi^FoxP3^+^ and CD25^low^FoxP3^+^ Tregs subsets in MDD patients (*p* = 0.001). Furthermore, the percentage of total Tregs (*p* = 0.005) and of their CD25^hi^FoxP3^+^ (*p* = 0.003) subset in the circulating CD4^+^T population in MDD patients were significantly higher than those found in HCs.

**Figure 3:**
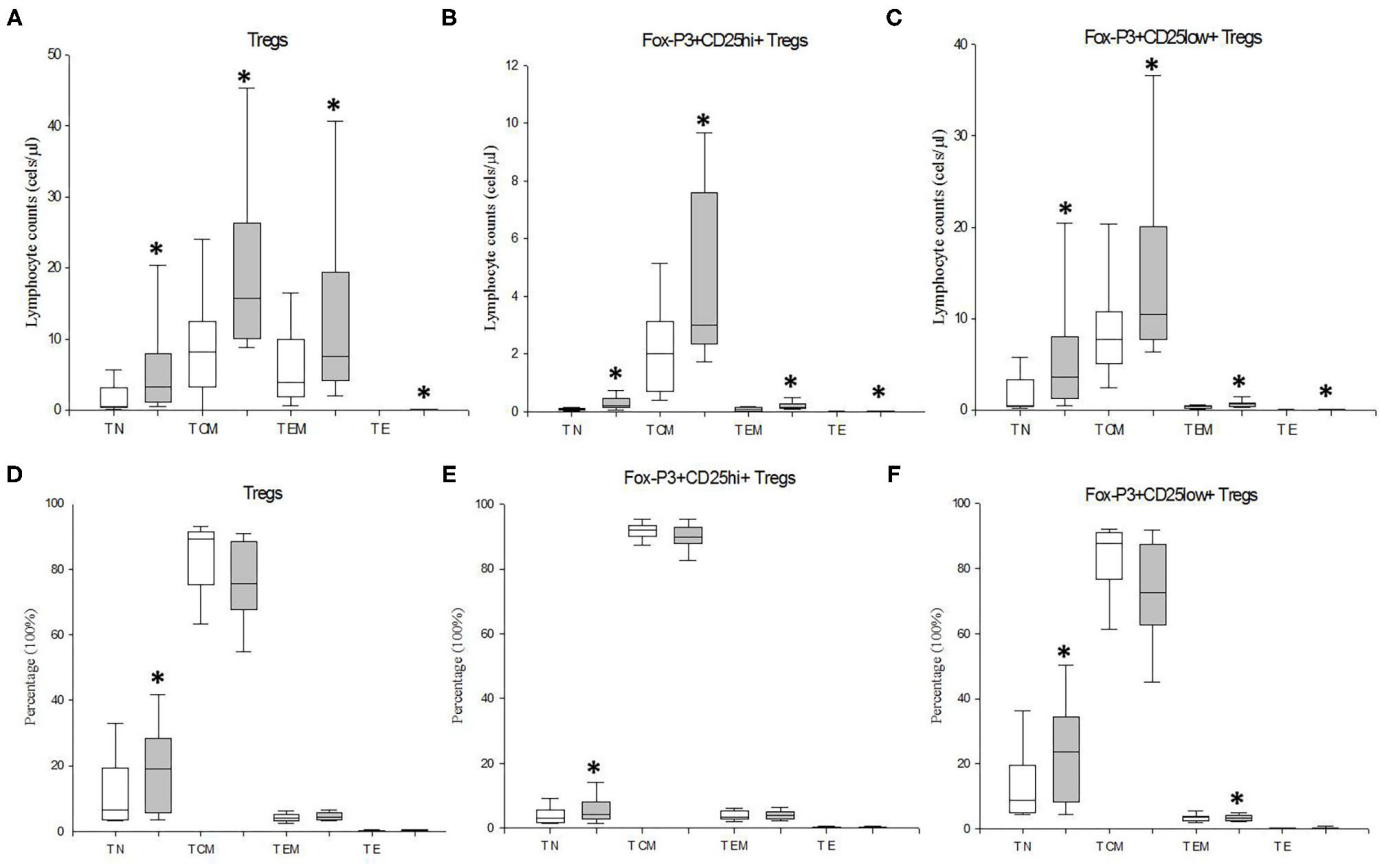
Absolute number and frequency of circulating total Tregs and their CD25^hi^FoxP3^+^ and CD25^low^FoxP3^+^ subsets at the T_N_, T_CM_ T_EM_, and T_E_ stages of CD4+ T lymphocyte differentiation/activation in MDD patients and healthy controls. Absolute number (cells/μl) (y axis) of Tregs lymphocytes and their CD25^hi^FoxP3^+^ and CD25^low^FoxP3^+^ subsets at the T_N_, T_CM_ T_EM_, and T_E_ stages of CD4+ T lymphocyte differentiation/activation in MDD patients (gray box plots) and healthy controls (white box plots) are shown in **(A–C)**, respectively. Frequency of the total Tregs and their CD25^hi^FoxP3^+^ and CD25^low^FoxP3^+^ subsets at the T_N_, T_CM_ T_EM_, and T_E_ stages of differentiation/activation in MDD patients (gray box plots) and controls (white box plots) are shown in **(D–F)**, respectively. The top of the rectangle indicates the third quartile, the horizontal line near the middle of the rectangle indicates the median, and the bottom of the rectangle indicates the first quartile. A vertical line extends from the top of the rectangle to indicate the maximum value, and another vertical line extends from the bottom of the rectangle to indicate the minimum value. *Significant difference between MDD and healthy controls for the indicated variable.

We also investigated the counts and distribution of Treg cells and their CD25^hi^FoxP3^+^ and CD25^low^FoxP3^+^ subsets at the T_N_, T_CM_ T_EM_, and T_E_ stages of CD4+ T lymphocyte differentiation/activation in MDD patients and HCs (Figure 3). Significant enhanced numbers of total and both Tregs subsets were found at the four stages of CD4+ T lymphocyte differentiation/activation in MDD patients (*p* < 0.001). We also found a redistribution of the Treg cells and their CD25^hi^FoxP3^+^ and CD25^low^FoxP3^+^ subsets along the four stages of activation/maturation. The percentages of CD25^+^FoxP3^+^ Treg cells (*p* = 0.024) and their CD25^hi^FoxP3^+^ (*p* = 0.01) and CD25^low^FoxP3^+^ subsets (*p* = 0.01) at T_N_ stage were significantly higher in MDD patients than those found in HCs.

We investigated the potential correlation between the patients' HDRS score and their circulating Treg cells and their CD25^hi^FoxP3^+^ and CD25^low^FoxP3^+^ subsets but, we did not find statistically significant correlations. Neither did we find statistically significant differences between the Tregs counts among the patients that suffered their first depressive episode and those with at least a previous episode. In addition, we did not find statistically significant differences neither with respect to patients with or without antipsychotic drugs nor men and women (Data not shown).

### Tregs From MDD Patients Show Increased CCR6 and CCR2, but Normal CCR5 Expression

**Figure 4:**
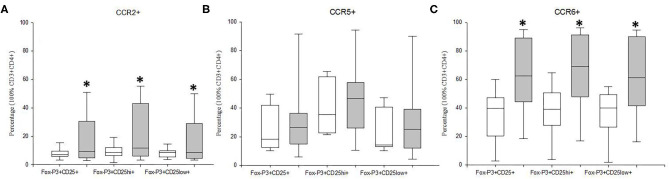
Frequency of CCR2, CCR5 and CCR6 expression by Tregs and their CD25^hi^FoxP3^+^ and CD25^low^FoxP3^+^ subsets in MDD patients and healthy controls, Frequencies of total Tregs and their CD25^hi^FoxP3^+^ and CD25^low^FoxP3^+^ subsets (y axis) in MDD patients (gray box plots) and healthy controls (white box plots) that express the CCR2 **(A)**, CCR5 **(B)**, and CCR6 **(C)**. The top of the rectangle indicates the third quartile, the horizontal line near the middle of the rectangle indicates the median, and the bottom of the rectangle indicates the first quartile. A vertical line extends from the top of the rectangle to indicate the maximum value, and another vertical line extends from the bottom of the rectangle to indicate the minimum value. *Significant difference between MDD and healthy controls for the indicated variable.

We studied the frequency of Tregs cells and of their CD25^hi^FoxP3^+^ and CD25^low^FoxP3^+^ subsets that express the CCR2, CCR5, and CCR6 chemoreceptors in MDD patients and HCs (Figure 4). We found that the percentages of the total Tregs and both subsets that expressed CCR2 and CCR6 were significantly higher in MDD patients than that in HCs (*p* < 0.05). However, there were not significant differences in the percentage of Tregs that express CCR5 between MDD patients and HCs.

### MDD Patients Show Increased IL10 Productions by Tregs

**Figure 5:**
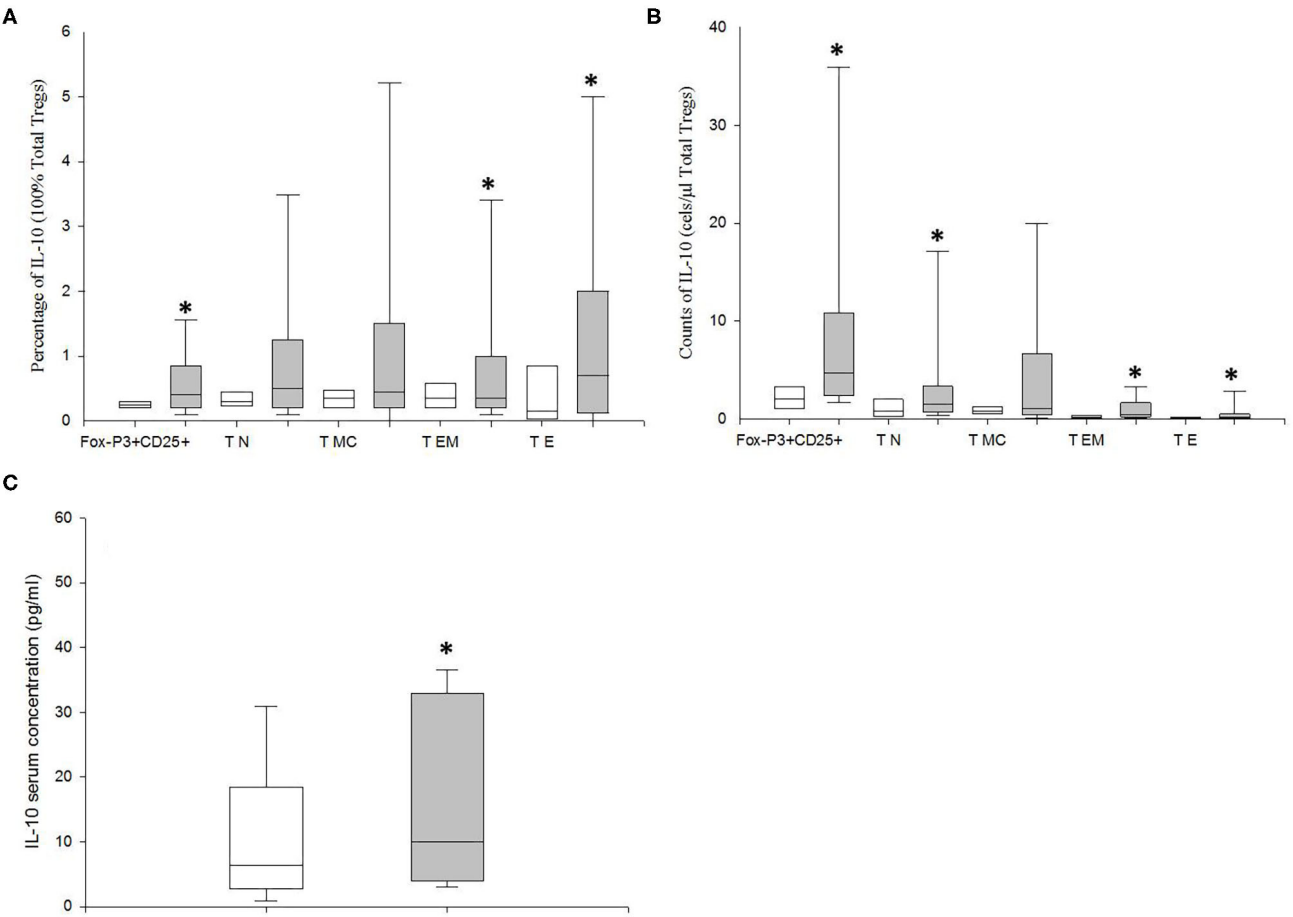
Frequency of IL-10 expression and number of circulating total Tregs and of those at the T_N_, T_CM_ T_EM_, and T_E_ stages of differentiation/activation able to produce this cytokine in MDD patients and healthy controls. Frequencies of total Tregs and of those at the T_N_, T_CM_ T_EM_, and T_E_ stages of differentiation/activation (y axis) in MDD patients (gray box plots) and healthy controls (white box plots) that express IL10 (x axis) after *in vitro* PMA (50 ng/ml) stimulation for 4 h in total **(A)**. Absolute number (cells/μl) (y axis) of circulating total Tregs and of those at the T_N_, T_CM_ T_EM_, and T_E_ stages of differentiation/activation in MDD patients (gray box plots) and healthy controls (white box plots) that are able to express the indicated cytokine (x axis) after *in vitro* PMA stimulation for 4 h **(B)**. Serum concentrations (pg/μl) (y axis) of IL-10 MDD patients (gray box plots) and healthy controls (white box plots) **(C)**. The top of the rectangle indicates the third quartile, the horizontal line near the middle of the rectangle indicates the median, and the bottom of the rectangle indicates the first quartile. A vertical line extends from the top of the rectangle to indicate the maximum value, and another vertical line extends from the bottom of the rectangle to indicate the minimum value. *Significant difference between MDD and healthy controls for the indicated variable.

We studied the intracellular expression of IL-10 in the Tregs from MDD patients and HCs after PMA stimulation. We found that the percentage of the total Tregs that expressed IL-10 in MDD patients was significantly higher than that in HCs (*p* = 0.02) (Figure 5). The increased percentage of Tregs expressing IL-10 in MDD patients was explained by the significantly enhanced percentages found in the T_EM_ and T_E_ differentiation/activation stages (*p* = 0.03 and *p* = 0.02, respectively). Next, in both groups of subjects, we calculated the potential number of circulating Tregs that could express IL-10 by multiplying their number by the percentage of cells that express the cytokine after PMA stimulation in both groups of subjects (Figure 5). We observed that the number of circulating Tregs that could express IL-10 was significantly increased in the MDD patients compared to that found in the HCs (*p* = 0.02).

We also measured the circulating levels of IL-10 in MDD patients and HCs (Figure 5). We found that MDD patients had significantly increased serum concentration of IL-10 compared to HCs (*p* = 0.04).

### Increased LBP Serum Levels Are Associated With a Reduction of Tregs in MDD Patients

**Figure 6:**
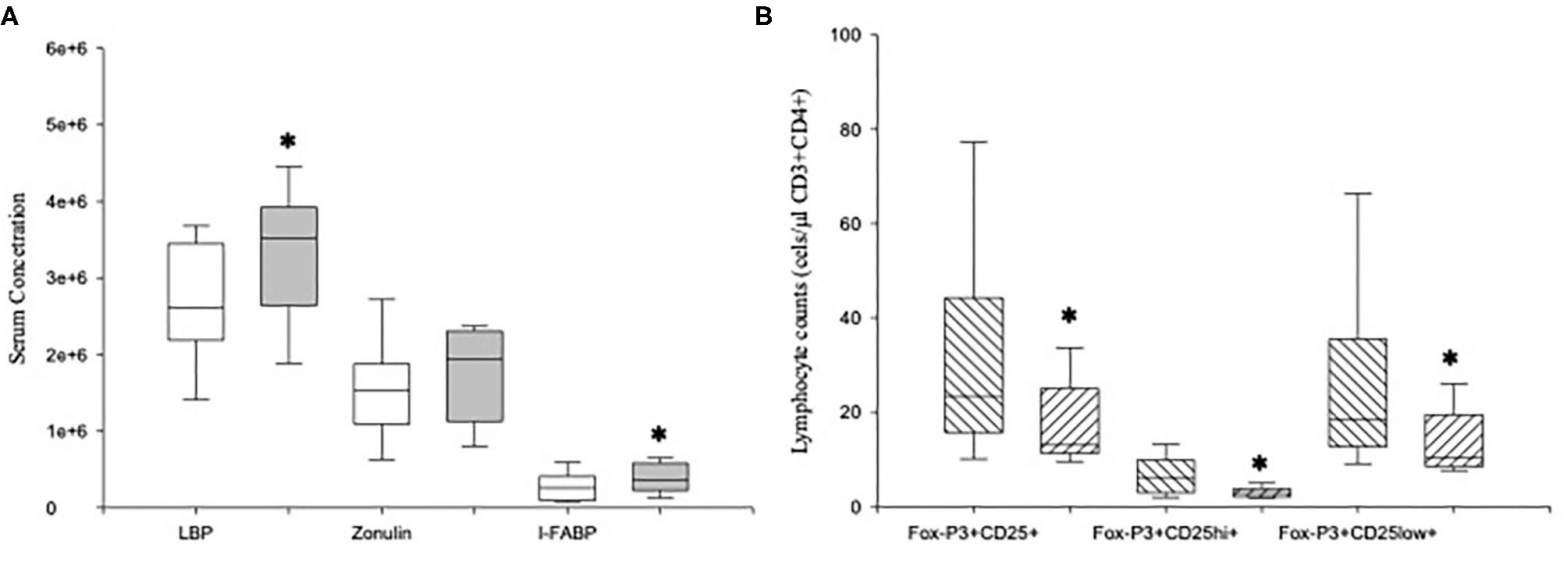
Serum levels of LBP, zonulin and I-FABP in MDD patients and absolute number of circulating Tregs and their CD25^hi^FoxP3^+^ and CD25^low^FoxP3^+^ subsets in high-LBP and normal-LBP MDD patients. Serum concentrations (y axis) of LBP (ng/ml), zonulin (mg/ml) and I-FABP (pg/ml) in MDD patients (gray box plots) and healthy controls (white box plots) **(A)**. Absolute number of circulating Tregs and their CD25^hi^FoxP3^+^ and CD25^low^FoxP3^+^ subsets (y axis) in high-LBP (ascending lined box plots) and normal-LBP (descending lined box plots) MDD patients subsets and healthy controls (white box plots) **(B)**. The top of the rectangle indicates the third quartile, the horizontal line near the middle of the rectangle indicates the median, and the bottom of the rectangle indicates the first quartile. A vertical line extends from the top of the rectangle to indicate the maximum value, and another vertical line extends from the bottom of the rectangle to indicate the minimum value. *Significant difference between MDD and healthy controls for the indicated variable.

We measured the serum concentrations of LBP, zonulin, and I-FABP in MDD patients and HCs (Figure 6). MDD patients showed significantly increased levels of LBP and I-FABP compared to those found in HCs (*p* = 0.04 and *p* = 0.01, respectively). We detected LBP, zonulin and I-FABP in all the serum from patients.

We found that 11 (high-LBP MDD) out of the 30 MDD patients had LBP levels higher than the 95th percentile of those found in HCs (Figure 6). In addition, we found that the counts of Tregs and of both CD25^hi^FoxP3^+^ and CD25^low^FoxP3^+^ Tregs subsets in MDD patients with high-LBP MDD were significantly lower than those found in normal-LBP patients (*p* = 0.003, *p* = 0.05, *p* < 0.001, respectively). Interestingly, there were not significant differences in the counts of Tregs and of both CD25^hi^FoxP3^+^ and CD25^low^FoxP3^+^ Tregs subsets between high-LBP MDD patients and HCs. However, we did not observe significant differences in the expression of IL-10 in the Tregs between both groups of MDD patients (data not shown).

## Discussion

In this paper, we have demonstrated that compared to healthy controls, MDD patients show a marked expansion of circulating Tregs and their CD25^high^FoxP3^+^ and CD25^low^FoxP3^+^ subsets through the different T_N_, T_E_, T_CM_, and T_EM_ stages of CD4+ T lymphocyte activation/differentiation. Tregs also show an increased frequency of cells that express CCR6 and CCR2. Furthermore, the percentage of CD25^+^FoxP3^+^ Tregs that produce IL-10 is also enhanced in MDD patients that concurrently have increased enhanced serum IL-10 levels. However, this Tregs expansion was blunted in MDD patients with gut barrier damage and increased bacterial translocation.

MDD is associated to a systemic inflammatory response that has been defined by the established increased concentrations of circulating proinflammatory cytokines and chemokines found in these patients (39). In addition, clinical findings and cellular studies support the knowledge of abnormal function of the natural and adaptative immune responses (14). The understanding of cellular mechanisms involved in the MDD immune system dysfunction remains elusive.

Tregs are a cornerstone of the immune system playing a critical role in the regulation of the activation and effector activity of cells of the innate and adaptive immune response (40). Tregs appear to be pivotal in the control and suppression of the immune responses against non-self and self-antigens (41). Our data show a clear increase in the number of Tregs and in its frequency in the CD4^+^ T lymphocyte circulating population in MDD patients. Circulating Tregs represent a versatile and dynamic cell population that is composed of different functionally heterogeneous subsets (42). In MDD patients, our data demonstrate an expansion of both effector CD25^hi^FoxP3^+^ and resting CD25^low^FoxP3^+^ Tregs subsets compared to those of the HCs supporting the observed increase in the numbers of total circulating Tregs. Conflicting results regarding the Tregs numbers have been previously described in MDD patients (28–30, 43). Several reasons that are not mutually exclusive may explain the heterogeneity in the numbers of circulating Tregs found in MDD patients. First, The differences in methodology for identification of Tregs may explain the observed discrepancies between studies. We have applied in this study a current stringent and precise cytometric strategy of Tregs characterization. Second, The clinical characteristics of the study samples. We have analyzed a homogenous population of patient with MDD with persistent symptomatology for an interval between 10 and 20 weeks. We included as controls a balanced sex, age, and BMI group of HCs from a similar epidemiological area. We studied patients with persistent MDD symptoms in spite of pharmacological treatment because we aimed to discover the relevance of the impact of the severe disease excluding those with rapid response to treatment. Furthermore, with this inclusion criteria, we avoid the reported increase in Tregs numbers during effective antidepressant treatment, whatever antidepressant is prescribed (44). To prevent potential interference of concomitant or previous disease and/or treatment with the immune system function, we applied precise exclusion criteria supporting the homogeneity of the population and the absence of potential causes of interference. Third. A new light of explanation of the heterogeneity in the numbers of circulating Tregs reported in MDD patients might be inferred from our finding of two groups of patients, defined by the serum high- and normal-LBP levels, with normal or increased counts of circulating Tregs, respectively. Differences in the frequency of subjects from both groups of patients in a cohort may explain discrepancies in the counts of the Tregs observed between studies. We did not find differences in the Tregs counts between the patients suffering their first episode and those with recurrent disease, which supports linking the expansion of these lymphocytes with the presence of MDD symptomatology.

The activation of Tregs driven by antigen stimulation promotes their progression from the naïve stage to the memory and effector stages characterized by differentiated functional and phenotype properties. Thus, in addition to the quantitative analysis of Tregs in MDD patients, we investigated their distribution along the different activation/differentiation stages. MDD patients show increased numbers of total Tregs and of both CD25^hi^FoxP3^+^ and CD25^low^FoxP3^+^ Treg subsets at the different T_N_, T_E_, T_CM_, and T_EM_ stages. These results agree with previous articles reporting increased percentage of memory Treg cells in the MDD patients, but the authors did not describe the other stages of Tregs activation/differentiation (30). We observed a preferential expansion of these Tregs populations at the T_N_ stage in MDD patients. Although, thymus-derived and peripherally derived Treg have been identified, our results do not favor the claimed impairment in Tregs generation in MDD patients (31, 43). Conversely, this finding suggests that MDD is associated to a host environment, favoring the cell progression to further activation/differentiation advanced T_CM_ T_EM_ and T_E_ stages (45, 46). The observed enhanced expression of IL-10 by Tregs from MDD patients also support the relevance of an environment promoting Tregs activation in these patients.

The pattern of CCR expression by Tregs is involved in the regulation of their homing and inflammatory response patterns. We have found that Tregs from MDD patients show increased frequency of cells that express CCR6 and CCR2. It has been reported than CCR2 and CCR 5 are necessary to ensure efficient homing of Treg to inflammatory tissue and migration toward the antigen site (25). These findings suggest that Tregs from MDD patients may have an anomalous pattern of tissue migration. The acquisition of homing receptor phenotypes probably occurs after the activation of periphery Tregs (23). Thus, the potential relevance of environment promoting abnormal Tregs activation in MDD may be also suggested.

Different mechanisms may be involved in the pathophysiology of the Treg abnormalities found in MDD patients. As previously discussed, the observed expansion of functionally active Tregs in approximately two thirds of the MDD patients might be favored by a promoting environment. The consequences of this Treg expansion may be relevant for the understanding of the pathogenesis of the disease. There is increasing evidence of the pathophysiological relevance of Treg heterogeneity and plasticity (46). CD25^low^FoxP3^+^ Tregs can lose FoxP3 expression and acquire effector Th cell function, such as the Th1 and Th17 phenotypes, under certain conditions, whereas CD25^hi^FoxP3^+^ Tregs are rather stable on mice. Furthermore, human Treg cells seem to be rather unstable. CD25^hi^FoxP3^+^ Treg cells may differentiate into Th17 producer cells in the presence of inflammatory cytokines (47). Former FoxP3^+^ T cells are also found to be particularly increased in inflamed tissues (48, 49). Thus, it is possible to speculate that a Th1 and Th17 plasticity of the increased numbers of Tregs found in MDD patients might occur and contribute to a systemic pro-inflammatory disbalance. Furthermore, the expansion of active Tregs with suppressor activity on the adaptive immune response may be involved in the pathogenesis of increased susceptibility to viral infections, reduced immune responses to vaccines and the slowed wound healing observed in MDD patients (12–14).

A third of MDD patients have not developed Treg expansion. In these patients, we found marked gut barrier damage and increased bacterial translocation. There is evidence that the microbiota influences the immune system and vice versa. More specifically, there are close interactions between the gut microbiota and the Tregs (50). The balance of effector lymphoid cells and Treg cells can have a profound influence on how the gut mucosa responds to stressors that elicit damage (51). Furthermore, MDD is associated with an enhanced intestinal permeability, or “leaky gut,” and increased bacterial translocation (52, 53). In this subset of MDD patients, we have confirmed enhanced I-FABP serum levels, a proven peripheral blood marker of gut barrier function (54). This abnormal intestinal mucosa barrier favors bacterial, including gram -, translocation. Circulating LPS promotes the hepatic synthesis of LBP, an acute phase reactant, and LPS-LBP complexes bind to CD14 on the monocyte membrane with a subsequent activation of the cell. In several clinical settings, the long- term (72 h) plasma levels of LBP induced by transient endotoxemia appear to better reflect long-term exposure to LPS than does measuring LPS itself (55). Of interest, LBP is just one of several markers of bacterial translocation. In agreement with previous reports, our findings show that as a group, MDD patients have increased LBP serum levels, with a third also showing a markedly high concentration of this bacterial translocation marker (56). Interestingly, we observed a significant reduction in the counts of Tregs and in both the CD25^hi^FoxP3^+^ and CD25^low^FoxP3^+^ Treg subsets in high-LBP MDD patients in comparison to those found in normal-LBP MDD patients. The clinical characteristics of the MDD patients included in this study exclude chronic viral or bacterial infections, as well as any existence of a concomitant or recent (at least 3 months prior to the study) bacterial infection. Thus, these results strengthen the connection between intestinal barrier dysfunction, bacterial translocation, and the immune-inflammatory system in MDD patients, with an expansion of activated monocytes in MDD patients (53). It is possible to suggest that the reduction of the expansion of Tregs might limit the gut migration of these cells. This potential reduction of Tregs of systemic origin could compromise the total number of intestinal Tregs (57). Treg restriction in the gut mucosa might favor gut barrier damage and the increased bacterial translocation found in this previously referenced third of MDD patients. Although there is no direct evidence of causality, our results support the relevance of the so-called gut-brain axis, linking gastrointestinal function and the immune system with the emotional and cognitive brain centers (58, 59).

This work has limitations. It is a cross sectional study and the course of the disease on natural Tregs need to be formally tested and replicated in longitudinal studies. The clinical homogeneity of the MDD patients studied combined with their very similar HDRS score, undermines the ability to study the potential association of the Treg expansion with the severity of the disease. Furthermore, the characteristics and size of the sample included in this pathophysiological study also limits the analysis of the potential association of the Tregs abnormalities with the clinical features of the disease. We have analyzed a homogenous population of MDD patients presenting persistent symptomatology for 10–20 weeks. However, all patients were medicated, and it might be a cofound. Furthermore, in this study, we were not able to control for additional factors that might also be associated with dysregulated cellular immunity, such as hormonal status, e.g., during menstrual cycle phases, physical activity, or other lifestyle- or environment-associated factors.

Taking these results together, two different MDD subsets can be established according to the analysis of circulating Tregs. Two thirds of them have an expansion of Tregs whereas the remaining third has normal Treg numbers, which were associated with severe gut barrier damage and increased bacterial translocation. These results support the heterogeneity of the mechanisms of immune-inflammatory dysfunction in MDD patients, as well as a need for the individualized biological study of patients for the development of new immunoregulatory therapeutic strategies.

## Data Availability Statement

The raw data supporting the conclusions of this article will be made available by the authors, without undue reservation.

## Ethics Statement

The studies involving human participants were reviewed and approved by Ethics committee of the University of Navarra and the Ethics committee of the Hospital Universitario Principe de Asturias. The patients/participants provided their written informed consent to participate in this study.

## Author Contributions

All authors contributed significantly to this work and have approved the manuscript content.

## Conflict of Interest

The authors declare that the research was conducted in the absence of any commercial or financial relationships that could be construed as a potential conflict of interest.
